# Manganese-Doped N-Hydroxyphthalimide-Derived Carbon Dots—Theranostics Applications in Experimental Breast Cancer Models

**DOI:** 10.3390/pharmaceutics13111982

**Published:** 2021-11-22

**Authors:** Adrian Tiron, Corneliu S. Stan, Gabriel Luta, Cristina M. Uritu, Irina-Cezara Vacarean-Trandafir, Gabriela D. Stanciu, Adina Coroaba, Crina E. Tiron

**Affiliations:** 1 TRANSCEND Centre, Regional Institute of Oncology, 2-4 General Henri Mathias Berthelot Street, 700483 Iasi, Romania; adrian.tiron@iroiasi.ro (A.T.); gabriel.luta@iroiasi.ro (G.L.); irina.trandafir@iroiasi.ro (I.-C.V.-T.); 2 Department of Natural and Synthetic Polymers, “Gheorghe Asachi” Technical University of Iasi, 71 Prof. dr. docent Dimitrie Mangeron Street, 700050 Iasi, Romania; stancs@tuiasi.ro; 3 Centre for Advanced Research and Development in Experimental Medicine (CEMEX), “Grigore T. Popa” University of Medicine and Pharmacy, 16 Universitatii Street, 700115 Iasi, Romania; gabriela-dumitrita.s@umfiasi.ro; 4 Centre of Advanced Research in Bionanoconjugates and Biopolymers, “Petru Poni” Institute of Macromolecular Chemistry, 41A Grigore Ghica Voda Alley, 700487 Iasi, Romania; adina.coroaba@icmpp.ro

**Keywords:** carbon dots, anti-cancer, MRI, nanomedicine, theranostic

## Abstract

Background: Theranostics, a novel concept in medicine, is based on the use of an agent for simultaneous diagnosis and treatment. Nanomaterials provide promising novel approaches to theranostics. Carbon Dots have been shown to exhibit anti-tumoral properties in various cancer models. The aim of the present study is to develop gadolinium, Fe^3+^, and Mn^2+^-doped N-hydroxyphthalimide-derived Carbon Dots. The resulted doped Carbon Dots should preserve the anti-tumoral properties while gaining magnetic resonance imaging properties. Methods: Normal and cancer cell lines have been treated with doped Carbon Dots, and the cell viability has been measured. The doped Carbon Dots that exhibited the most prominent anti-tumoral effect accompanied by the lowest toxicity have been further in vivo tested. Magnetic resonance imaging evaluates both in vitro and in vivo the possibility of using doped Carbon Dots as a contrast agent. Results: According to the results obtained from both the in vitro and in vivo experimental models used in our study, Mn^2+^-doped Carbon Dots (Mn-CDs-NHF) exhibit anti-tumoral properties, do not significantly impair the cell viability of normal cells, and reduce lung metastasis and the volume of mammary primary tumors while allowing magnetic resonance imaging. Conclusions: Our findings prove that Mn-CDs-NHF can be used as theranostics agents in pre-clinical models.

## 1. Introduction

Due to the fact that cancer still remains a major worldwide health problem despite the latest advances in understanding cancer biology and developing new tools for early detection and treatments, researchers are looking for new ways to improve drug delivery and treatments toward being less invasive as well as having fewer side effects and higher efficiency [1].

Different technologies, such as nanomedicine, have opened up new opportunities for the treatments in different diseases. Nanocomposites play an important role in drug release and biodistribution in various types of diseases. For example, they could improve the release of chemotherapeutic drugs in the therapy of certain cancer types [2]. Nanoparticles (NPs) have been widely employed in the past few years in order to deliver therapeutic drugs more effectively to different targeted tissues [3,4]. Several studies indicate that nanomaterials-based delivery systems suppress in vivo tumor growth [5,6,7].

In this context, Carbon Dots-based materials have gained attention in the scientific community due to their physical and chemical properties, which make them one of the most promising tools for drug delivery and imaging [8,9]. Cytotoxicity studies on different cell lines demonstrate the lower toxicity of Carbon Dots even at high concentrations. Moreover, Carbon Dots have recently been proven in vitro and in vivo to exhibit anti-tumoral properties in various cancer models [10,11,12,13,14]. Our previous work demonstrated in vitro and in vivo the anti-tumoral properties of N-Hydroxyphthalimide-derived Carbon Dots (CDs-NHF) in a breast cancer model [15]. In another paper, we have shown that although CDs-NHF alone do not impair U87 glioblastoma cell line viability, combining lower doses of key pathways inhibitors with lower CDs-NHF doses significantly reduced cell viability [16].

Despite the outstanding advances in medical imaging for diagnosis, magnetic resonance imaging (MRI) remains one of the most valuable techniques for in vivo investigations, due to its non-invasive nature in the absence of ionizing radiation, which may provide both anatomical and functional data [17]. The majority of MRI contrast probes are based on gadolinium (Gd^3+^). However, due to the fact that Gd^3+^ is associated with different side effects such as nephrogenic system fibrosis, new alternatives are necessary [18,19].

Manganese-based nanoparticles (Mn-based NPs) represent promising alternatives due to their lower toxicity compared with Gd^3+^ [20,21]. Additionally, there are no data to report any pre-clinical data regarding the in vitro and in vivo toxicity of Mn-based NPs, making them a viable alternative to Gd^3+^ [22,23].

Apoptosis or programed cell death is a regulatory system having a crucial role in controlling cell proliferation, tissue homeostasis, and removing cells that are no longer needed. The dysregulation of apoptosis mechanisms contributes to human diseases such as cancer. Molecules responsible for apoptosis have offered new ways to develop solutions in order to increase cancer cell death. Mitochondria play an important role in apoptotic cell death. Mitochondria dysfunction leads to apoptosis [24,25].

The cytokine interleukin-6 (IL-6) plays a crucial role in both normal human physiology and diseases. The findings demonstrate that IL-6 promotes the development of breast [26], colorectal [27], pancreatic [28], and skin [29] cancers. IL-6 signaling has been shown to play an important role in tumor progression and metastasis dissemination in a different tumor type [30]. High circulating levels of IL-6 have been reported in patients with different types of cancers such as breast [31], colorectal [32], ovarian [33], renal [34], non-small-cell lung cancer (NSCLC) [35], and head and neck [36], and circulating IL-6 levels are reported to be increased in patients with recurrent tumors [37].

In the present study, we aim to develop a theranostics agent that combines the anti-tumoral properties of CDs-NHF with enhanced imaging properties, which are doubled by low toxicity of Mn. For this, we have developed doped CDs-NHF with Gd^3+^, Mn^2+^, or Fe^3+^. Using an MRI in vitro model, we have investigated the anti-tumoral effects of doped CDs-NHF as well as the imaging properties. The most promising candidate, Mn-CDs-NHF, has been further investigated in an in vivo 4T1 murine breast cancer model in order to evaluate its theranostics potential. Additionally, we have also aimed to explore the effect of the Mn-CDs-NHF on lung metastases due to the fact that lungs represent the primary metastasis site of the 4T1 cell line.

## 2. Materials and Methods

### 2.1. Preparation and Investigation of the Doped CDs-NHF

The preparation of the doped CDs-NHF was performed according to the procedure and the experimental setup mentioned in our previous work [38]. N-Hydroxyphthalimide (97%) (NHF) and anhydrous MnCl_2_ were sourced from Merck Chemicals. High-purity water and reagent grade ethanol (EtOH) were used for all preparation stages. Briefly, the first stage involves the preparation of a Mn(II)–NHF complex at a ½ metal/ligand ratio, according to the following reaction:

MnCl_2_ + 2(C_8_H_5_NO_3_) → [Mn(C_8_H_4_NO_3_)_2_(H_2_O)x] + HCl↑.

The complexation reaction undergoes at 35–40 °C, under stirring for about 24 h in a water/EtOH (60/40% volume ratio) medium. The precipitate is collected, washed twice with water, and further freeze dried. In the second stage, the prepared complex is thermally processed through pyrolysis in the same conditions and process parameters using the previously mentioned experimental setup [38]. The resulted aqueous dispersion of Mn-CDs-NHF was purified and dimensionally selected through centrifugation and further freeze dried to obtain a fine powder which was re-dispersed in a convenient solvent according to the testing requirements.

The mean zeta potential of the samples was evaluated by DelsaNano C Submicron Particle Size Analyzer from Beckman Coulter Inc. Light source: Dual 30 mW laser diodes as 658 nm. For zeta potential, the instrument uses electrophoretic light scattering (ELS) to measure the zeta potential, which determines the electrophoretic movement of charged particles under an applied electric field.

### 2.2. Cell Cultures and Cell Viability Assay

Murine breast cancer 4T1cells (American Type Culture Collection, Rockville, MD, USA, ATCC), a generous gift from James Lorens (Bergen Bio AS, Bergen, Norway), were cultured in RPMI-1640 media supplemented with 10% fetal bovine serum (Sigma-Aldrich^®^, St. Louis, MO, USA). Normal human mammary epithelial (HMLE) cells (ATCC), a generous gift from James Lorens (Bergen Bio AS, Bergen, Norway), were cultured in DMEM/F12 supplemented with 5% horse serum, 20 ng/mL EGF, 10 ug/mL insulin, 0.5 ng/mL hydrocortisone, 10 ng/mL cholera toxin, and 1% Pen/Strep. Cells were seeded into a 96-well flat-bottom tissue culture plate at a density of 3000 cells/well and allowed to adhere to the plate by incubating at 37 °C under 5% CO2 overnight. Following overnight cell attachment, the cells were incubated with the tested doped CDs-NHF at 5% concentrations (50 µg CDs-NHF/mL) for 72 h. For each doped CDs-NHF, we have tested the amount of compound of that contained 50 µg CDs-NHF/mL.

For cell viability assessment, we used the CellTiter-Blue^®^ Cell Viability Assay (Promega). After each of the 72 h treatment time periods, 50 μL of cell viability solution was added to each well, and the plate was reincubated for 4 h before luminescence recording using a multiplate microplate reader (FilterMax F5, Sunnyvale, CA, USA).

### 2.3. Mouse Strain and Animal Care

The experiments were approved by Ethical Committee of the “Grigore T. Popa” University of Medicine and Pharmacy of Iasi and were performed in accordance with the European legislation on the protection of animals used for scientific purpose and with authorization from the National Sanitary Veterinary and Food Safety Authority (no. 17/09.04.2020). Female BALB/c mice (6–8 weeks old; Cantacuzino Institute, Bucharest, Romania) were used. The mice were housed in the animal facility of the CEMEX, “Grigore T. Popa” University of Medicine and Pharmacy, Iasi; in individually ventilated cages (IVCs) in a climate-controlled: 20 ± 4° Celsius, 50 ± 5% relative humidity, and 12 h light/dark cycles containing shaving bedding material with regular rodent chow and water ad libitum.

### 2.4. Mammary Fat Pad Spontaneous Metastasis Model

4T1 mouse breast carcinoma cells were suspended in RPMI medium/Matrigel (1:1) (1 × 10^6^ in 50 μL) and injected into the mammary pad of female BALB/c mice under depth anesthesia, as previously described [15]. At two weeks post tumor cells inoculation, mice had been started to be treated via intraperitoneal injection (twice per week) with 10% (100 µg/mL) Mn-CDs-NHF (*n* = 6) or Gadovist (*n* = 6) for 3 weeks. CD-NHF concentration is relative to mice blood volume and represents one of the previously tested concentrations [15].

At the end of the testing period, the animals were euthanized (neck dislocation under deep anesthesia), and primary tumors and various organs (liver, kidneys, lungs, spleen) were collected. Half of each primary tumor and half of each lung were immediately suspended in RNA Save (Biological Industries, New Haven, CT, USA) and stored at −80 °C until use. The other half from each primary tumor and each lung were preserved in 10% paraformaldehyde (Sigma-Aldrich) for further analysis.

### 2.5. In Vitro MRI Investigations

The in vitro experiments were planned to evaluate the potential of the new prepared doped CDs-NHF as MRI contrast agents by determining the values of longitudinal and transversal relaxivities, r1 and r2, respectively, which were calculated from the corresponding T1 and T2 relaxation times. From each compound, a 5 mg/mL stock solution was prepared by dissolving 20 mg of powder in 4 mL of PBS 0.01 M, at physiological pH (7.4). The samples for MRI scanning were prepared by dispersing 10 ÷ 60 µL stock solutions in 10 mL of 1% agarose gel freshly prepared, at about 50 °C to obtain six concentrations for each tested compound, as follows: 0.005, 0.01, 0.015, 0.02, 0.025, and 0.03 mg/mL.

The T1 and T2 measurements were performed using a 1 Tesla instrument for small animals (nanoScan PET-MRI, Mediso LTD), having B0 magnetic field shimming and coil calibration at a water proton frequency. The glass vials comprising the samples to be analyzed were positioned horizontally on the rat body acquisition bed in the center of the coil field of view. T1 relaxation times were determined by a two-point estimation method [39] through a 2D spoiled gradient echo sequence (T1 GRE), while for T2 measurements, we have used a multiecho 2D standard spin echo sequence (T2 SE).

### 2.6. In Vivo MRI Investigations

Two groups of tumor-bearing mice (females), comprising 6 mice per group, were investigated once per week by MRI scanning: one group named “Treated”, which has received Mn-CDs-NHF as a teranostic agent via i.p. administration and another group named “Control”, receiving only Gadovist, which is a commercial contrast agent. Both Mn-CDs-NHF and Gadovist were administered by i.p. injection 2 h prior to the MRI examination.

Fasted mice (not more than 6 h) were anesthetized using an isoflurane delivery system connected to a transparent chamber at a concentration of 3.5–4% isoflurane in a mixture of air and oxygen. After induction, the mice were transferred to the scanner bed of the nanoScan PET/MRI (Mediso^®^, Budapest, Hungary), and maintenance anesthesia was adjusted to 2%. The animals were subjected to MRI scanning using T1GRE sequence, coronal, and axial planes. This sequence was selected due to the short investigation time: 22 min for both planes. The parameters were as follows: TR = 13.1; TE = 3.8/2.2; FA = 30°; NSA = 4; slice thickness = 1 mm. The raw imaging data were processed using Carimas software (v. 2.10), which allowed the calculation including the tumor volume, apart from 2D images for each section.

### 2.7. RNA Extraction, cDNA Synthesis, and Quantitative RT-PCR (qRT-PCR) Analysis

For in vitro investigations, total RNA was isolated from the control and treated samples (experimental design) using TRIzol reagent (Thermo Fisher Scientific, Waltham, MA, USA) according to the manufacturer’s protocol. Briefly, approximately 10^7^ cells per sample were mixed with 1 mL of Trizol before RNA was separated from DNA and proteins by adding 200 μL chloroform. Then, the total RNA was precipitated using 500 μL of isopropanol. The RNA pellet was washed afterwards with 1 mL of 75% ethanol solution and finally eluted in 11.5 μL ultrapure sterile water (Biomol, United States Biological).

For in vivo investigations, small fragments (≈1 mm/1.5 mm) of tissue were cut/scraped and diced with a sterile scalpel and homogenized prior to the RNA extraction. Briefly, tissue was homogenized in 1 mL of Trizol (Thermo Fisher Scientific) before the RNA was separated from DNA and proteins by adding 200 μL chloroform. Then, the total RNA was precipitated using 500 μL of isopropanol. The RNA pellet was washed afterwards with 1 mL of 75% ethanol solution and finally eluted in 11.5 μL ultrapure sterile water (Biomol, United States Biological).

A list of the primers used in this study, as well as the species, gene symbol, gene name, UniGene ID, and primer sequences is in Supplementary Table S1.

### 2.8. Immunofluorescence (IF)

IF was performed on 4 µm thick sections of formalin-fixed and paraffin-embedded tissues. Sections were deparaffinized and rehydrated; then, they were stained for detecting mitochondria in the fixed cells (IraZolve-Mito, Rezolve Scientific, Adelaide, Australia) or the apoptotic cells (DeadEnd Fluorometric TUNEL System, Promega) according to the manufacturer’s recommendations.

### 2.9. Statistics

GraphPad Prism 6 (San Diego, CA, USA) software was used for statistical analysis. Grouped analyses were performed using one-way ANOVA for cell viability investigations and paired Student’s *t*-test for other statistical analysis. Quantitative data for statistical analysis were expressed as mean ± SEM (shown as error bar). Significance was established for *p* < 0.05.

## 3. Results

### 3.1. Characterization of Mn-CDs-NHF

The structural analysis showed a typical Mn^2+^-doped CDs-NHF (Mn-CDs-NHF) structure, which consists mostly of a carbonaceous core with a surface decorated with different remnant functional groups. The thermal destruction of the Mn(II)-NHF complex through the controlled pyrolytic process resulted in the formation of this graphitic-like core with a variety of functional groups attached on its surface, as previously studied [40,41]. XPS analysis revealed the formation of the carbonaceous core along with the relative concentrations of the various functional groups. Therefore, the recorded wide scan spectrum of Mn-CDs-NHF revealed the overall atomic and mass concentrations as presented in the Supplementary Table S2. As it could be noted, the preponderant carbon presence sustains the formation of the carbonaceous core, while the presence of O, N, and Mn indicates the presence of the various remnant functional groups. The high-resolution spectra recorded for the Mn-CDs-NHF are presented in Supplementary Figure S1a–d together with the relative concentrations of the attached remnant groups.

Mn-CDs-NHF dispersed in water exhibit the same agglomeration tendency as observed in the case of non-doped CDs-NHF presented in our previous work [40], which was most likely as a result of the interactions between the functional groups from the surface that favor clustering. As it could be observed in Supplementary Figure S2, the cluster sizes are mainly located within the 250–400 nm range. These results are sustained by the SEM investigation (Supplementary Figure S3), where it is clearly visible that the Mn-CDs-NHF organize in clusters within the range of 100–300 nm.

The surface charge was determined by measuring the zeta potential of the synthetized CDs. The zeta potential value is determined by the stability of particles. Particles with a high zeta potential (either negative or positive) are electrostatically stable, while the particles with low zeta potentials tend to aggregate within a short period of time. The positive values of the zeta potential reflect the presence of a positively charged environment around the CDs. Having in mind the previously presented facts, the analyzed samples, CDs-NHF and Mn-CDs-NHF, which have positive values of zeta potential, confirm that both un-doped and doped CDs have positively charged surroundings. Moreover, it is obvious that the CDs-NHF sample possesses higher colloidal stability, having a mean zeta potential value of +16.59 mV, whereas the Mn-CDs-NHF sample possesses lower colloidal stability. The mean zeta potential value in this case is +6.87 mV, meaning that this sample tends to aggregate. The zeta potential measurement for each sample was acquired in triplicate, and the obtained values (Supplementary Table S3) are plotted in Supplementary Figure S4 as the mean zeta potential with SD.

### 3.2. Cell Viability Assay

**Figure 1:**
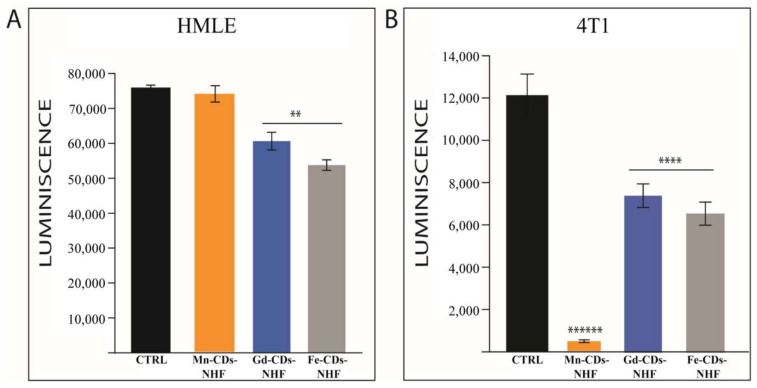
Effects of doped CDs-NHF on cell viability. (**A**). Normal cell line HMLE; (**B**). Cancer cell line 4T1. ** *p* < 0.05, **** *p* < 0.0005, ****** *p* < 0.00005.

Our previous results [15] demonstrated that 5% CDs-NHF (50 µg CDs-NHF/mL) treatment resulted in the decreased migration, invasion, and viability/proliferation of tested cancer cell lines without significantly affecting the tested normal cell line. As a result of doping, it may be possible that the obtained doped CDs-NHF exhibits altered anti-tumoral properties than the previously tested, non-doped CDs-NHF. Therefore, assessing cell viability is the first step in investigating the theranostics application of doped CDs-NHF (Figure 1).

The cell viability data demonstrate that gadolinium (Gd^3+^)-doped CDs-NHF (Gd-CDs-NHF) and Fe^3+^-doped CDs-NHF (Fe-CDs-NHF) significantly reduced the cell viability in both tested normal (HMLE) (Figure 1A) and cancer (4T1) cell lines (Figure 1B). Mn-CDs-NHF had the strongest effect on cancer cell line when compared to the control (untreated group) and the other two doped CDs-NHF, without significantly impairing the normal cell line viability.

### 3.3. In Vitro MRI Imaging

**Figure 2:**
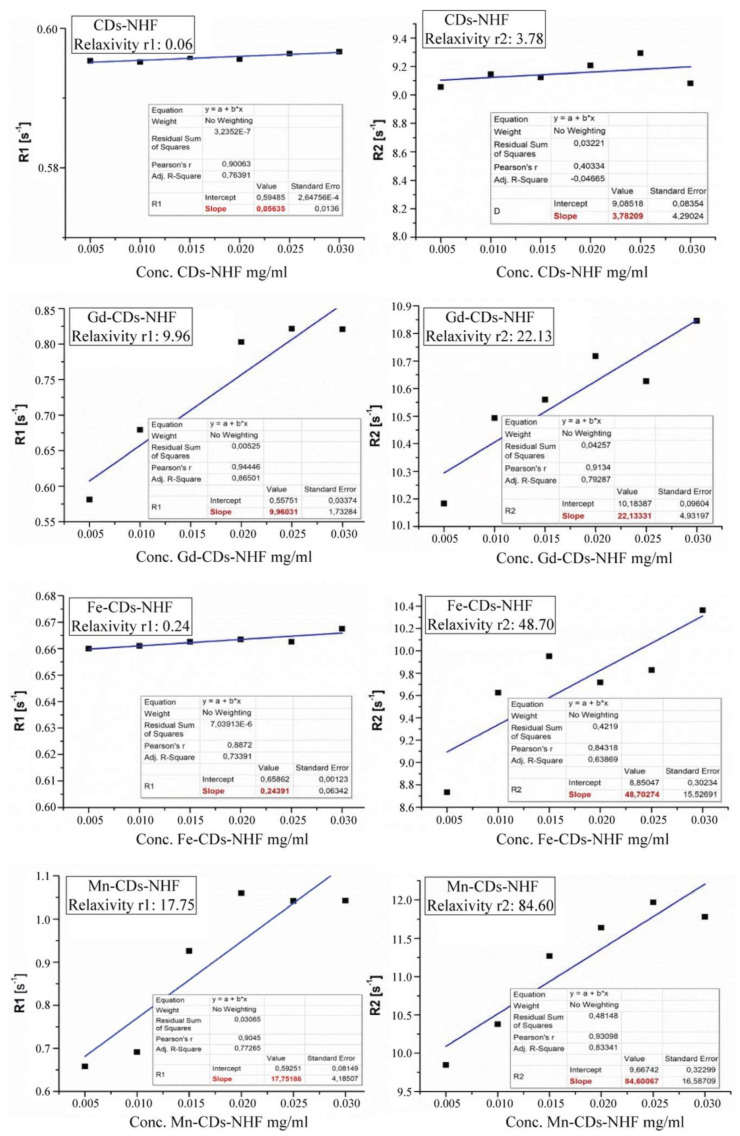
The r1 and r2 relaxivities of CD-NHF (negative control), Gd-CD-NHF (positive control), Fe-CD-NHF, and Mn-CDs-NHF.

In order to develop theranostics-doped CDs-NHF, in addition to their anti-tumoral properties, the imaging capabilities must be investigated. In our work, we have tested doped CDs-NHF as contrast agents in MRI (Figure 2).

The r1 and r2 relaxivity values represent the slope of the linear regression of T1 and T2 relaxation rates (1/T1 and 1/T2, respectively) in the range of the product concentrations we have considered. The relaxivity values indicate the T1/T2 shortening ability of the tested compounds with a direct impact on the MRI contrast. According to the r1/r2 values obtained, as it can be observed from Figure 2, the Mn-CDs-NHF best meets the feature of an MRI contrast agent. In addition, in the case of Fe-CDs-NHF, the ability of the trivalent ion to provide NMR contrast in T2 sequences is confirmed by a high r2 value of 48.70 mg^−1^·mL·s^−1^, while r1 is insignificant to provide MRI contrast, of only 0.24 mg^−1^·mL·s^−1^. Since gadolinium is the only ion with clinical applicability to enhance the MRI contrast, it was considered in this study as a positive control, which was bound into the Gd-CDs-NHF in a similar manner to the rest of metal included in the prepared compounds. In such an environment, the Gd^3+^ ion caused relaxivity values of r1 and r2 of 9.96 and 22.13 mg^−1^·mL·s^−1^, respectively, suggesting a good ability to enhance the MRI contrast, especially in T2 sequences. Quite different is the case of Mn-CDs-NHF product, which although it has a relaxivity r2 significantly higher than all other materials tested, of 88.60 mg^−1^·mL·s^−1^, it also presents a noticeable value for r1, of 17.75 mg^−1^·mL·^s−1^, which is close to the r2 value of Gd-CDs-NHF. In such circumstances, we can infer that the Mn-CDs-NHF product is able to be successfully applied as a contrast agent in both T1 and T2 sequences, depending on what is pursued in that study. The in vitro imaging data using T1 and T2 MRI sequences are presented in Supplementary Figure S5.

Due to the fact that Mn-CDs-NHF exhibited the lowest impact on normal cell viability and the most effective impact on cancer cell viability, doubled by the fact that it can be successfully used as MRI contrast agent, we further investigate the Mn-CDs-NHF theranostics applications.

### 3.4. In Vivo MRI Imaging

**Figure 3:**
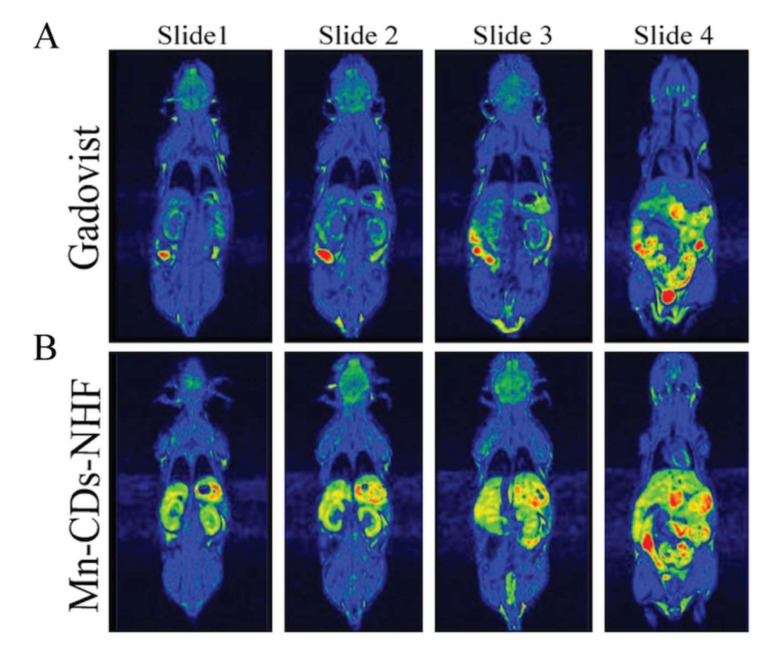
MRI comparative images of healthy mice highlighting the ability of the new compound, Mn-CDs-NHF as an MRI contrast agent (**B**) as compared to Gadovist, which is a commercial MRI contrast agent (**A**). Images are obtained using the same sequences, which were performed at 2 h after i.p. administration.

Prior to accomplishing the MRI scans on breast cancer animal models, the effect of the selected compound (Mn-CDs-NHF) as a contrast agent on healthy mice was studied by comparing the resulting images with those obtained using Gadovist MRI images (Figure 3). The same conditions and scanning parameters were met as in the groups of tumor-bearing animals.

An MRI signal is much more intense in the case of the group that received the suspension of Mn-CDs-NHF in comparison with the group receiving Gadovist. There is also a very obvious uptake of the Mn^2+^-based compound, which is predominantly in the liver, kidneys, peritoneal cavity, and heart and much less in the brain or other regions located distant from the injection site. The images are displayed in rainbow color visualization to show as suggestive as possible the difference in MRI contrast between different tissues, while the color intensities in all the recorded images were normalized using the same brightness level in all images so that they could be compared.

**Figure 4:**
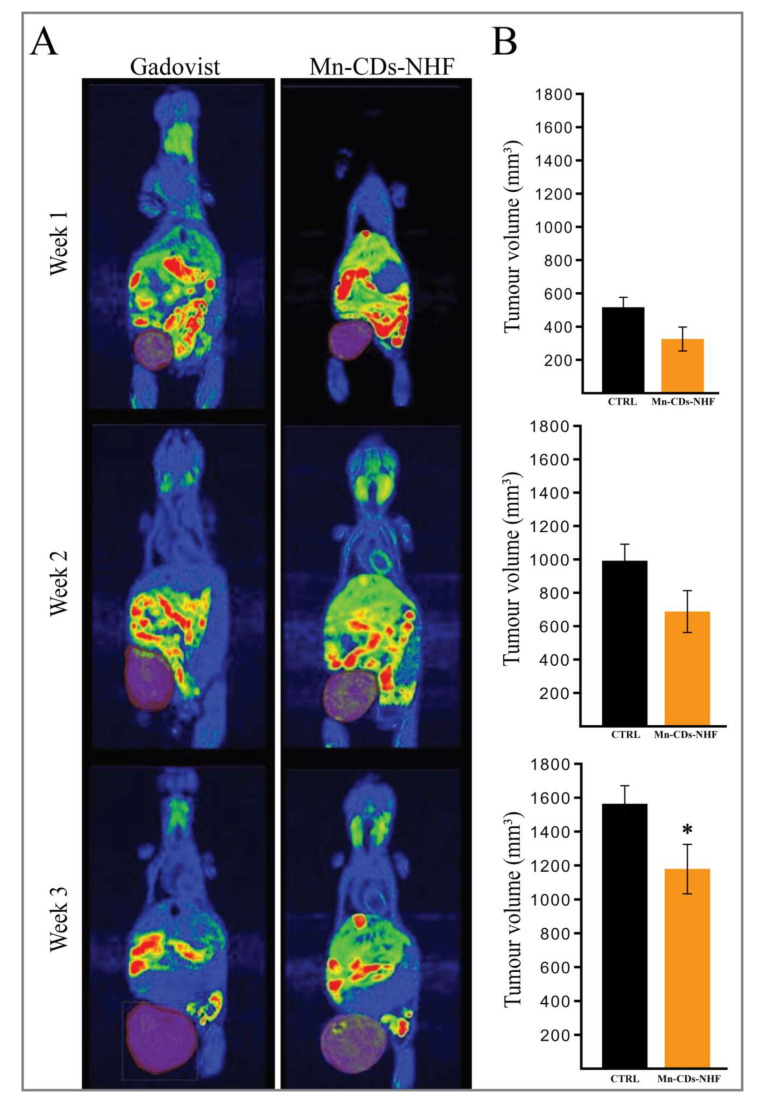
Representative MRI images and scorization of primary tumor volume. (**A**). Weekly images of tumor-bearing mice treated with Gadovist or Mn-CDs-NH. (**B**): Scorization of primary tumor volume for each treated group. * *p* < 0.05.

Representative weekly MRI images of tumor-bearing mice are presented in Figure 4, while the weekly evaluations of each tumor-bearing animal from both groups are presented in Supplementary Figures S6 and S7.

In addition to the improved MRI contrast capabilities of Mn-CDs-NHF relatively to the commercial agent Gadovist, it is also essential to notice that when compared with the control group (Figure 4A_Gadovist), the volume of the primary tumors is reduced in Mn-CDs-NHF treated group (Figure 4_Mn-CDs-NHF), as summarized in Figure 4B. These data demonstrate that the proposed teranostic agent, Mn-CDs-NHF, fulfill at the same time the role of increasing the MRI contrast as well the anti-tumor features.

### 3.5. Evaluation of Lung Metastases

**Figure 5:**
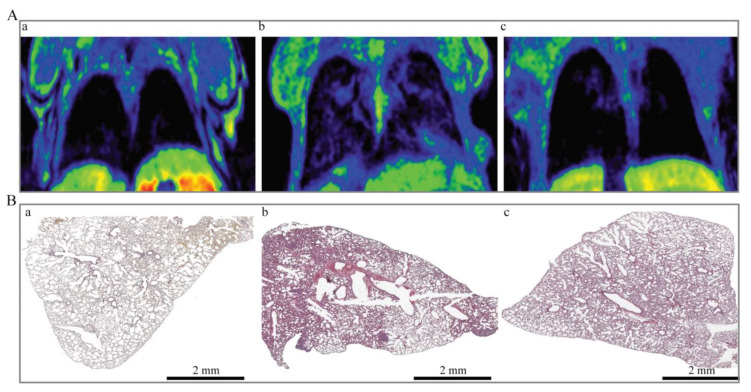
Metastatic investigation of lungs. (**A**) MRI images, (**B**) HE staining. (**a**) Healthy mice; (**b**) Tumor-bearing mice treated with Gadovist; (**c**) Tumor-bearing mice treated with Mn-CDs-NHF. Individual pictures acquired at 10× were used to rebuild the image of the lung specimens.

In clinical practice, the primary tumor is surgically removed, and metastases and local recurrence are usually responsible for the cancer recurrence. As the lung metastases are very common in the case of the 4T1 tumor cells, which could be highlighted by MRI, we also imaged the animals for the presence of lung metastases (Figure 5).

MRI imaging of thoracic cavity in the third week shows newly formed tissue, suggesting the presence of lung metastases. According to the MRI data, the prevalence of lung metastases was higher in the control group (Figure 5(Ab)) relatively to the Mn-CDs-NHF-treated group (Figure 5(Ac)). These data correlate with the results of hematoxylin and eosin (HE) staining of lung paraffin-embedded specimens (Figure 5B). The HE data of Mn-CDs-NHF (Figure 5(Bc))-treated group show a reduced number and size of lung metastases relative to the control group (Figure 5(Bb)) and an overall more normal resembling architecture of lung tissue, with less thickened alveolar walls (Figure 5(Ba)).

### 3.6. Mitochondria Evaluation in Paraffin-Embedded Lung Tissue

**Figure 6:**
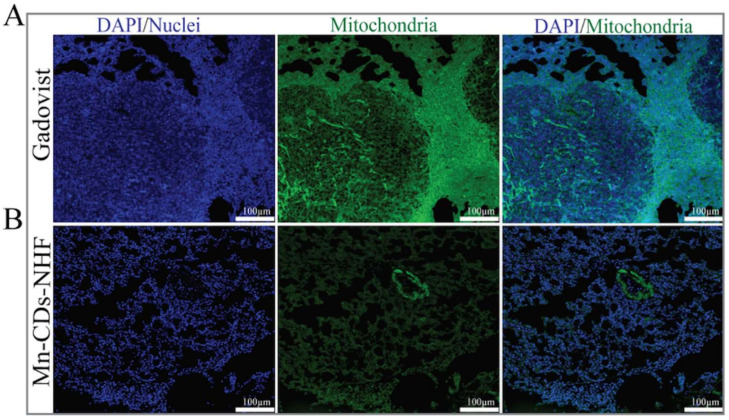
Immunofluorescence staining of mitochondria in paraffin-embedded murine lung specimens. Pictures acquired at 20×.

Our previous in vitro data showed reduced mitochondrial activity upon CDs-NHF treatment [42]. The new data (Figure 6) demonstrate that doping the CDs-NHF with Mn^2+^ do not affect the ability of CDs-NHF to reduce the number of mitochondria in cancer cells as assessed in paraffin-embedded lung tissue.

### 3.7. TUNEL Visualization of Apoptotic Cells in Lung Metastases

**Figure 7:**
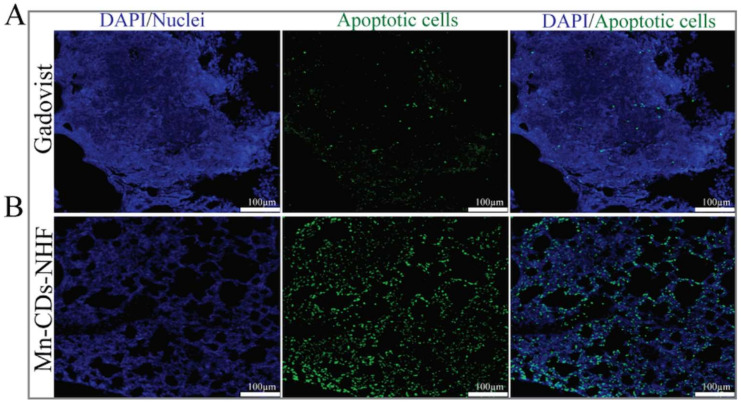
Immunofluorescence staining of apoptotic cells in paraffin embedded murine lung specimens. Pictures acquired at 20×.

The growth of the primary tumor, as well of metastases, is driven by cell proliferation and cell death. In our previous in vivo investigations [15], we have found that Ki67, a marker of cell proliferation, is decreased after CDs-NHF treatment. However, we did not investigate the apoptosis in the mouse tissue. In current work, we deployed TUNEL investigation for showing the apoptotic cells (Figure 7).

Our new data show only a few apoptotic cells in the control group (Figure 7A). The number of apoptotic cells is massively increased in the lung metastases of the Mn-CDs-NHF treated group (Figure 7B). These data sustain the in vitro cell viability data, which demonstrated reduced cell viability in the Mn-CDs-NHF-treated group relative to the Gadovist-treated group.

### 3.8. IL-6 qRT-PCR Investigations

**Figure 8:**
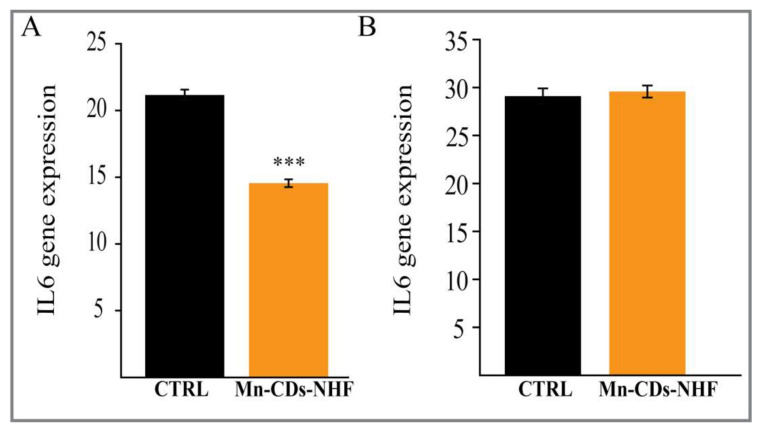
IL-6 mRNA expression in cell culture (**A**) and murine lung samples (**B**) *** *p* < 0.005.

Interleukin 6 (IL-6), a pro-inflammatory cytokine, mediates pro-survival signals in cancer and inflammations. Our in vitro investigation shows that the levels of IL-6 mRNA in cancer cell treated with Mn-CDs-NHF are significantly reduced relative to Gadovist-treated cells (Figure 8A).

However, we failed to observe these changes in lung specimens (Figure 8B), which was probably due to the dilution of IL-6 mRNA expressed by cancer cells from metastases with IL-6 mRNA expression from various normal cells.

## 4. Discussion

The increase in the morbidity and mortality of cancer cases put pressure on the scientists to find new tools for diagnostic and treatments. The detection of cancer at early stages can have a massive impact on cancer treatment and patient status. Developing agents with theranostics properties, both therapy and imaging, will improve early cancer detection and overall cancer management. The importance of theranostic agents in the diagnosis and therapy of cancer has been recently reviewed [43,44,45]. Since the widely used contrast agent, gadolinium, presents various side effects, we aimed to develop theranostic agents by doping CDs-NHF with ions that present lower toxicity. Previously, we have demonstrated the anti-tumoral properties of non-doped CDs-NHF in various models [15,16,42]. In our new experimental investigations, we decide to combine the anti-tumoral properties of CDs-NHF with MRI imaging capabilities of Gd^3+^, Fe^3+^, or Mn^2+^.

In a recent investigation [46], 24 h incubation of Fe(III)-doped Carbon Dots significantly reduces the cell viability of A549 lung cancer cells at concentrations ranging between 12.5 and 200 µg/mL. Our results indicate that after 72 h exposure of 50 µg/mL Fe-CDs-NHF, in addition to cancer cells, the viability of normal cells is impaired. Moreover, the cellular uptake of Carbon Dots reached its peak at 4 h and decreased after 12 h of incubation [46].

In another recent study [47], Carbon Dots decorated with quaternary ammonium groups were incorporated into the cytosol of mouse NIH/3T3 fibroblast cells from the concentration of 50 μg/mL and accumulated in the perinuclear area up to 200 μg/mL. The first sign of Carbon Dots inside the nucleus occurred at 300 μg/mL, and all NIH/3T3 cells had filled nuclei and nucleoli after exposure to 400 μg/mL. Moreover, the authors reported that tested concentrations (50–400 µg/mL) do not evoke notable DNA damage and do not impair NIH/3T3 cell viability. This was not the situation in the case of L929 mouse fibroblast cells where concentrations of 50–100 μg/mL did not cause viability impairment but higher concentrations induce cell dead without to target the nucleus. These data endorse the variety of impact on normal and cancer cells of the 50 µg/mL doped CDs-NHF we have tested.

Our in vitro findings showed that Mn-CDs-NHF exert a significant pro-apoptotic effect on cancer cell line without exhibiting a substantial impact on the normal cell line that we have tested. The other two doped CDs-NHF we have tested, Gd-CDs-NHF and Fe-CDs-NHF, significantly decreased the cell viability of the normal cell line. Moreover, the impact of Gd-CDs-NHF and Fe-CDs-NHF on cancer cell viability was lower relative to Mn-CDs-NHF, which indicates that doping CDs-NHF with Gd^3+^ or Fe^3+^ may reduce the anti-tumoral properties of CDs-NHF. The effect of Gd-CDs-NHF and Fe-CDs-NHF on both cell lines indicates that these two doped CDs-NHF exert a certain degree of toxicity. In addition to the in vitro data, the number of apoptotic cells is increased in the lung metastases in the Mn-CDs-NHF-treated group when compared with the classical MRI contrast agent, Gadovist. Even more, the decreased expression of the mitochondria staining in the Mn-CDs-NHF-treated group further sustains the anti-tumoral properties of Mn^2+^-doped CDs-NHF.

As we demonstrated that doping CDs-NHF with Mn^2+^ preserves the anti-tumoral properties of CDs-NHF, we have investigated next the MRI imaging application, which is the second characteristic of a theranostics agent. Both the in vitro cell line investigations and in vivo data from healthy mice demonstrate that the MRI signals of Mn^2+^ from Mn-CDs-NHF fulfill the second condition required for Mn-CDs-NHF to be used as a theranostics agent in experimental models. In tumor-bearing animal investigations, the lung metastases from the control group are well developed, and MRI imaging of the Mn-CDs-NHF group demonstrates that treatment with Mn^2+^-doped CDs-NHF reduces the primary tumor volume. These data are in line with our newly reported in vitro pro-apoptotic effect of Mn^2+^-doped CDs-NHF and with our previous reported data that showed that non-doped CDs-NHF treatment reduces the Ki67cell proliferation marker at the level of lung metastases [15]. The reduction of tumor volume requires time, becoming significant after three weeks of treatment administration and is a consequence of both the impairment of cell proliferation and the pro-apoptotic effect of Mn-CDs-NHF.

The MRI signal intensity of the liver, kidney and lung is enhanced in the Mn-CDs-NHF treated group relatively to healthy untreated mice, while the signal intensity of the brain and heart are at similar levels (Supplementary Figure S8). In week two, the MRI signal is relatively homogenously distributed in the entire primary tumors. In the third week, the MRI signal appears to be unevenly distributed; some areas have higher intensity, while other have lower intensity. The MRI signal distribution in the third week suggests the presence of larger necrotic areas in the primary tumors. MRI signal intensity is not different between the tumor core and tumor periphery in week two, but it becomes significant in week three (Supplementary Figure S9).

Altogether, our experimental findings demonstrate the theranostics properties of Mn-CDs-NHF. In comparison to Gd^3+^-based compounds (Gd-CDs-NHF and Gadovist), Mn-CDs-NHF exhibited lower toxic effects on normal cells, anti-tumoral properties, and a stronger MRI signal.

## Data Availability

The data presented in this study will be openly available upon publication.

## Supplementary Materials

The following are available online at https://www.mdpi.com/1999-4923/13/11/1982/s1, Table S1: List of the primers used in this study, species, gene symbol, gene name, NCBI accession number, mRNA reference sequence and primer sequences, Table S2: Overall atomic and mass concentrations recorded for the Mn-CDs-NHF, Table S3: Zeta potential results for CDs-NHF and Mn-CDs-NHF, Figure S1: High-resolution XPS spectra recorded for the Mn-CDs-NHF, Figure S2: Size distribution of the prepared Mn-CDs-NHF, Figure S3: SEM image recorded for the prepared Mn-CDs-NHF, Figure S4. Graphical representation of mean zeta potential with SD for CDs-NHF and Mn-CDs-NHF, Figure S5: The imaging data obtained by in vitro MRI scanning, Figure S6: Weekly evaluations by MRI of each tumor-bearing animal treated with Gadovist, Figure S7: Weekly evaluations by MRI of each tumor-bearing animal treated with Mn-CDs-NHF, Figure S8: In vivo tissue distribution of Mn-CDs-NHF, Figure S9: Quantification of MRI signal intensity in primary tumors.
