# Supplementary material for: Manganese-Doped N-Hydroxyphthalimide-Derived Carbon Dots—Theranostics Applications in Experimental Breast Cancer Models
Source: Pharmaceutics. 2021 Nov 22;13(11):1982. doi: 10.3390/pharmaceutics13111982 (PMC8674762; doi:10.3390/pharmaceutics13111982)
Supplement: Supplementary file 1 [file pharmaceutics-13-01982-s001.zip › pharmaceutics-1451703-supplementary.pdf]

pharmaceutics

Supplementary Materials: Manganese Doped N-Hydroxyphthalimide Derived Carbon Dots – Theranostics Applications in Experimental Breast Cancer Models

Adrian Tiron, Corneliu S. Stan, Gabriel Luta, Cristina Mariana Uritu, Irina-Cezara Vacarean-Trandafir, Gabriela Dumitrita Stanciu, Adina Coroaba and Crina Elena Tiron

qRT-PCR

The RNA yields and A260/280 ratios were measured by NanoDrop 2000 spectrophotometer (Thermo Fisher Scientific) with the OD260/OD280 ratio of all RNA samples varying from 1.81 to 1.99 and OD260/OD280 up to 2. The RNA was stored at -20˚C until reverse transcription in cDNA was performed. For each sample, 4 µl of total RNA were reverse transcribed into complementary DNA (cDNA) with the GoScript Reverse Transcription System (Promega) using random primers (0.5 µg/reaction), following the manufacturer's instructions using the Corbett CGI-96 Palm-Cycler Thermal Cycler equipment. The mixture was heated for 5 minutes at 70º C and then immediately placed on ice for another 5 minutes. To each tube, 4 µl of reaction buffer (5X), 1 µl of nucleotide mix and reverse transcriptase enzyme each, 0.5 µl of recombinant RNasin ribonuclease inhibitor, 1.5 µl MgCl2 and 7 µl of nuclease-free water were added to a final volume of 20 µl. Reactions were incubated at 25º C for 5 minutes, 42º C for one hour and 70º C for 15 minutes. cDNA was finally diluted to 20 ng/µl with nuclease-free water and was either immediately used or stored at -20º C until further use.

For the analysis, a Prime Time qPCR assay (IDT – Integrated DNA Technologies) was designed with premixed primers and probe (TaqMan) using the primer sequences with the corresponding gene references detailed in Table 1. Two housekeeping genes were used in this experiment: glyceraldehyde-3-phosphate dehydrogenase (GAPDH) and β2-microglobulin (B2M) and the amplifications were carried out in a Cobas z 480 (Roche) real-time PCR analyzer using 96-PCR-microplates (Roche). The qRT-PCR reaction mixture included 10 µl of Prime-Time gene expression Master Mix (2X) (IDT – Integrated DNA Technologies), 1 µl of Prime-Time qPCR Assay (20X, primers and probe) (IDT – Integrated DNA Technologies), 5 µl of cDNA template and 4 µl of Nuclease Free Water in a total volume of 20 µl for each reaction.

Table S1. List of the primers used in this study, species, gene symbol, gene name, NCBI accession number, mRNA reference sequence and primer sequences.

Species	Gene symbol	Gene name	NCBI reference sequence/mRNA referene sequence	Primer sequences
Homo Sapiens	GAPDH	Glyceraldehyde-3-phosphate dehydrogenase	NC_000012.12/NM_001256799.3	F: 5'-GGGGCTCTCCAGAACATCAT-3'<br>R: 5'-AAGTGGTCGTTGAGGGCAAT-3'

Homo Sapiens	IL-6	Interleukin 6, transcript variant 1	NC_000007.14/NM_000600.5	F: 5'-TTCGGTCCAGTTGCCTTCTC-3'<br>R: 5'-GAGGTGAGTGGCTGTCTGTG-3'
Mus Musculus	GAPDH	Glyceraldehyde-3-phosphate dehydrogenase	NM_008084/ Mm.436562	F: 5'- AATGGTGAAGGTCGGTGTG -3'<br>R: 5'- GTGGAGTCATACTGGAACATGTAG -3'
Mus Musculus	B2M	β2-microglobulin	NM_009735/ Mm.163132	F: 5'- TGGTCTTTCTGGTGCTTGTC -3'<br>R: 5'- GGGTGGAACTGTGTTACGTAG -3'
Mus Musculus	IL-6	Interleukin 6	NM_031168/ Mm.101909	F: 5'- ATACAAAGAAATGATGGATGCTACC -3'<br>R: 5'- CTTTCTTGTTATCTTTTAAGTTGTTCTTCA -3'

Preparation and investigation of the doped CDs-NHF

XPS analysis was performed on a KRATOS Axis Nova, using AlKa radiation with a 20 mA current and 15 kV voltage. The incident X-ray beam was focused on a 0.7 mm x 0.3 mm area. Wide XPS spectra were collected in the range of -10 to 1200 eV with a resolution of 1 eV and a pass energy of 160 eV while the high resolution spectra were collected using a pass energy of 20 eV and a step size of 0.1 eV. Dimensional analysis and size distribution of the Mn-CDs-NHF dispersed in water was performed on a Coulter Beckman Delsa Nano. SEM images were recorded on a Carl Zeiss NEON 40EsB with thermal Schottky field emission emitter and accelerated Ga ions column, working at 20KV acceleration voltage, beam resolution: 1.1÷2.5 nm. The samples were deposited from a diluted EtOH solution.

The zeta potential measurements were taken at 25 °C, and the final result is an average of three measurements. The analysis mode used the Smoluchowski equation. Each sample was dispersed in ultrapure water to obtain the desired concentrations (1 mg/mL) with a final volume of 2 mL. Zeta measurements were recorded using a Flow Cell module with the following software settings: sampling time: 400 μs, correlation channel: 512, cell constant: 70 1/cm, accumulation times: 50 (ten accumulations in five different points), scattering angle: 15°, correlation method: TD, pinhole: 100 μm, diluent: water, refractive index: 1.3328, viscosity: 0.8878 cP, dielectric constant: 78.3, Lorentz fitting: 1 peak. The obtained results were processed with Delsa Nano Software Version 3.73 from Beckman Coulter Inc and plotted with Microsoft Excel 2019.

Table S2. Overall atomic and mass concentrations recorded for the Mn-CDs-NHF.

	**Atomic conc. [%]**	**Mass conc. [%]**
Mn 2p	2.50	9.52
O 1s	23.79	26.42

N 1s	4.77	4.63
C 1s	68.94	59.42

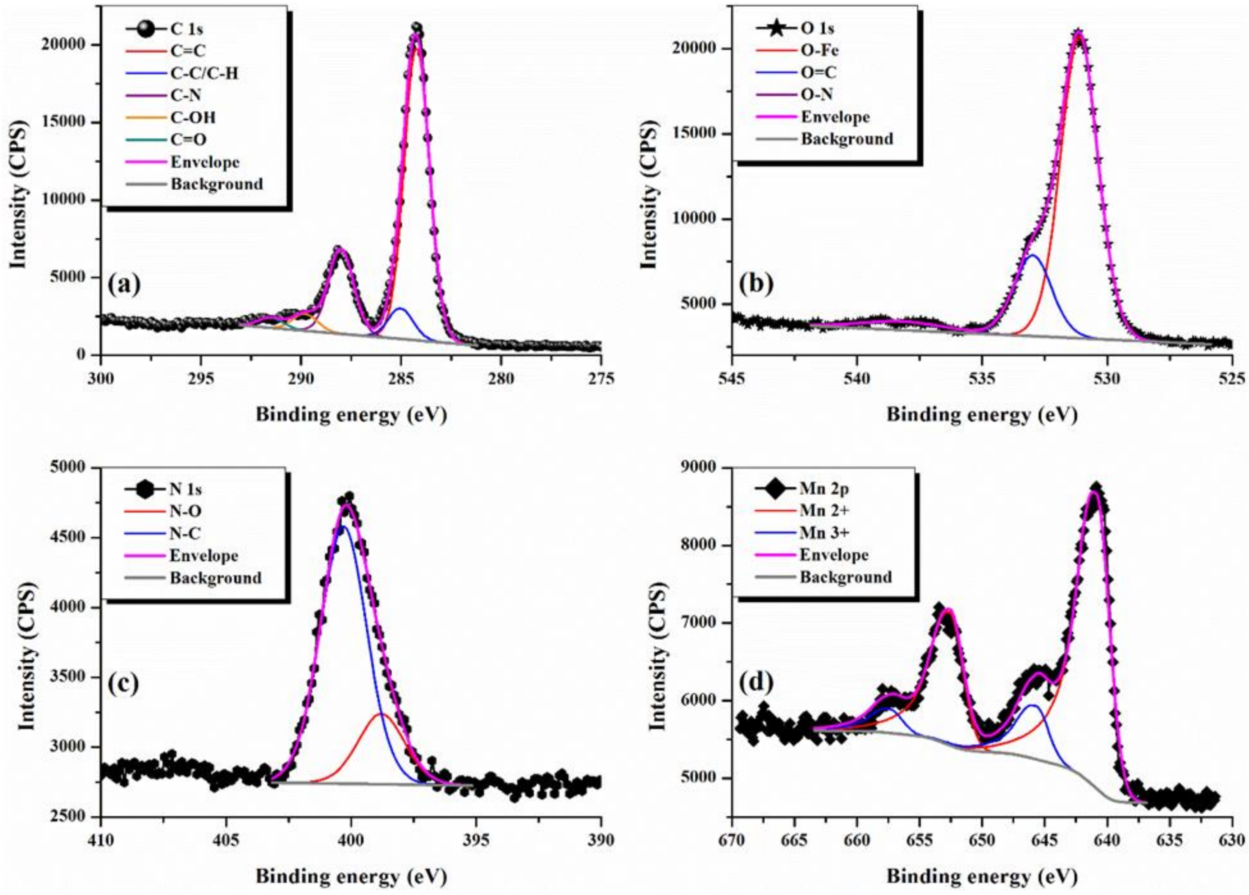

Figure S1. High resolution XPS spectra recorded for the Mn(II) doped Carbon Dots (a) C1s; (b) O1s; (c) N1s; (d) Mn2p

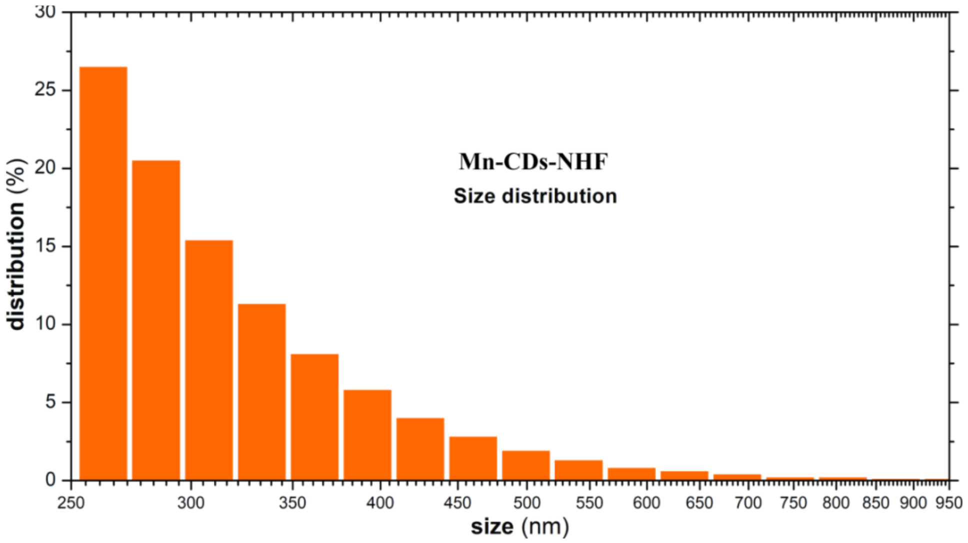

Figure S2. Size distribution of the prepared Mn-CDs-NHF

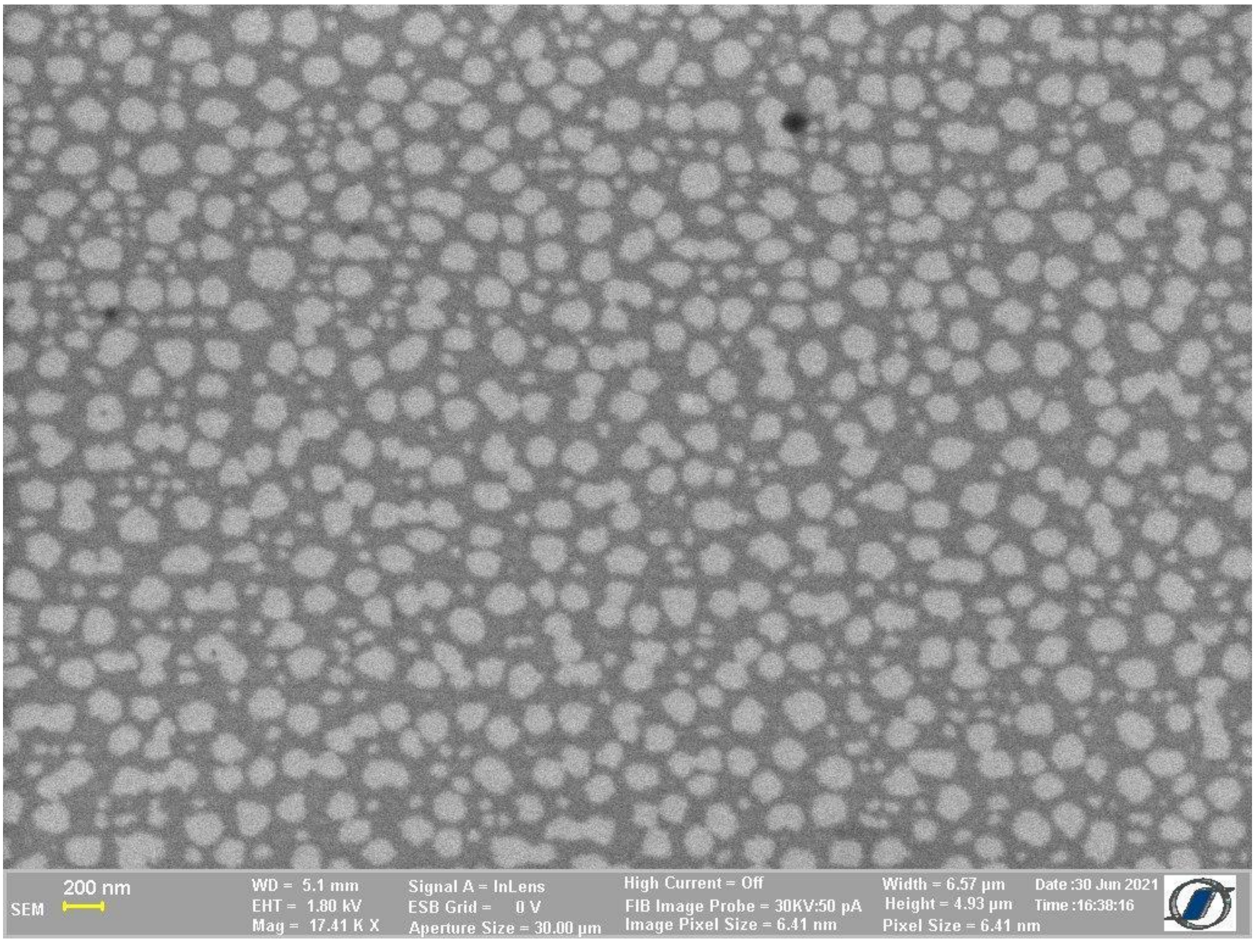

Figure S3. SEM image recorded for the prepared Mn-CDs-NHF.

Table S3. Zeta potential results for CDs-NHF and Mn-CDs-NHF

Sample	Conc. (mg/mL)	Solvent	Final volume (mL)	Meas. rep.	Zeta potential (mV)	Mobility x 10^{-5} (cm^2/Vs)	Conductivity (mS/cm)
**CDs-NHF**	1	mQ water	2	1	16.44	12.82	0.4967
				2	16.71	13.03	0.4930
				3	16.61	12.95	0.4852
				**Average**	**16.59**	**12.93**	**0.4916**
				**SD**	**0.1115**	**0.0865**	**0.0048**
**Mn-CDs-NHF**	1	mQ water	2	1	6.99	5.449	0.4123
				2	6.86	5.346	0.4106
				3	6.76	5.275	0.4083
				**Average**	**6.87**	**5.357**	**0.4104**
				**SD**	**0.0942**	**0.0714**	**0.0016**

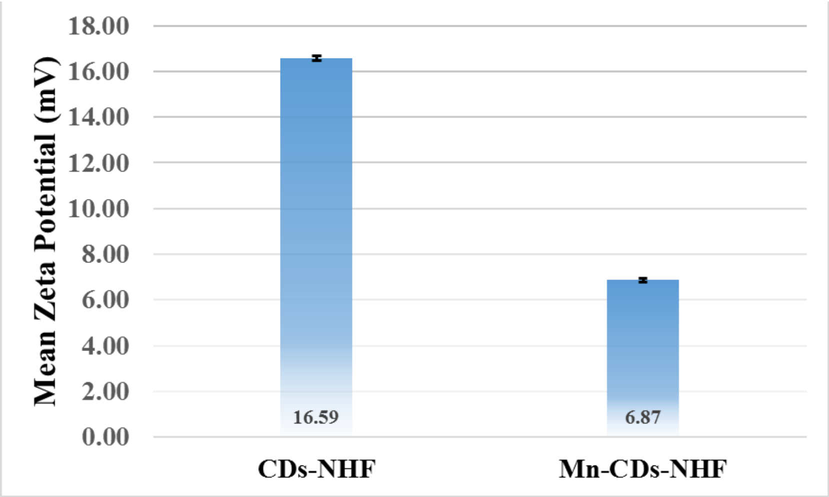

Figure S4. Graphical representation of mean Zeta potential with SD for CDs-NHF and Mn-CDs-NHF

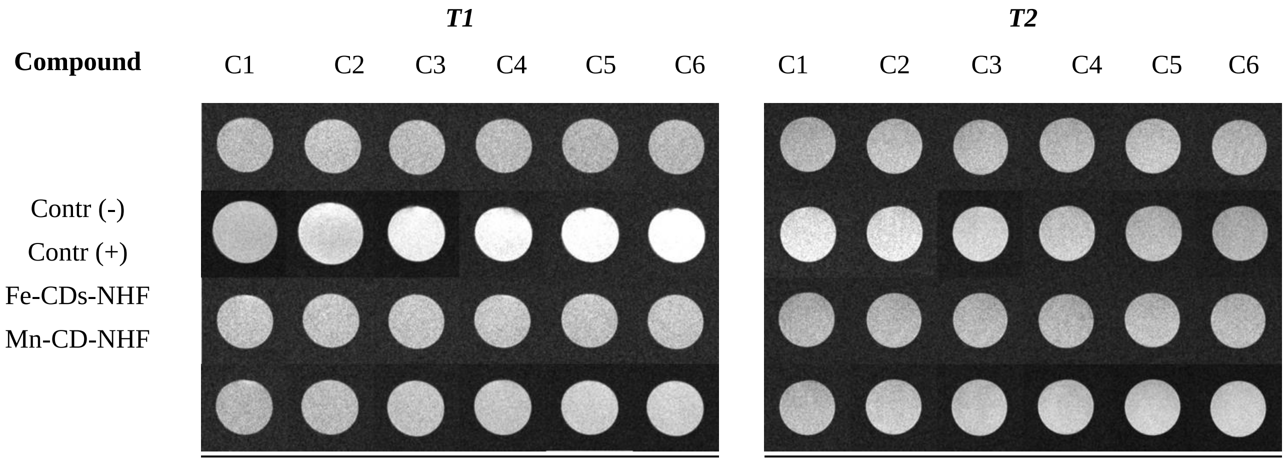

Figure S5. The imaging data obtained by in vitro MRI scanning of CDs-NHF (Contr (-)), Gd-CDs-NHF (Contr (+)), Fe-CDs-NHF and Mn-CDs-NHF using T1 and T2 MRI sequences, grouped in the two panels, left and right, respectively. The T1 main parameters were as follows: FA = 10 and 60°, TR = 167 ms, TE = 3.8 ms, NSA = 2, receiver bandwidth = 100 Hz/pixel, slice thickness = 5 mm, gap = 1 mm, in-plane resolution = 0.2mm, dummy cycles = 20 (TR – repetition time, TE – echo time, FA – flip angle). The T2 parameters were: TR = 400 ms, TE = 20, 40, 80, 120 ms, NSA = 2, receiver bandwidth = 100 Hz/pixel, slice thickness = 4 mm, gap = 1 mm, in-plane resolution = 0.2 mm.

To estimate the T1 relaxation times, the following calculation formula was considered:

$$\ln\left[\frac{(I_1 sin\theta_2 - I_2 sin\theta_1)}{(I_1 sin\theta_2 cos\theta_1 - I_1 sin\theta_2 cos\theta_1)}\right] = \frac{-TR}{T_1}$$

where I_1, I_2 are the signal intensities collected for circular regions of interest (ROI) of 10 mm diameter drawn inside the sample, θ_1 and θ_2 – flip angles, of 10° and 60°, respectively. T2 values were calculated based on the following formula:

$$I = A \cdot e^{\frac{TE}{T2}}$$

where I and A are the mean signal intensity in the ROI, and the initial signal intensity, respectively.

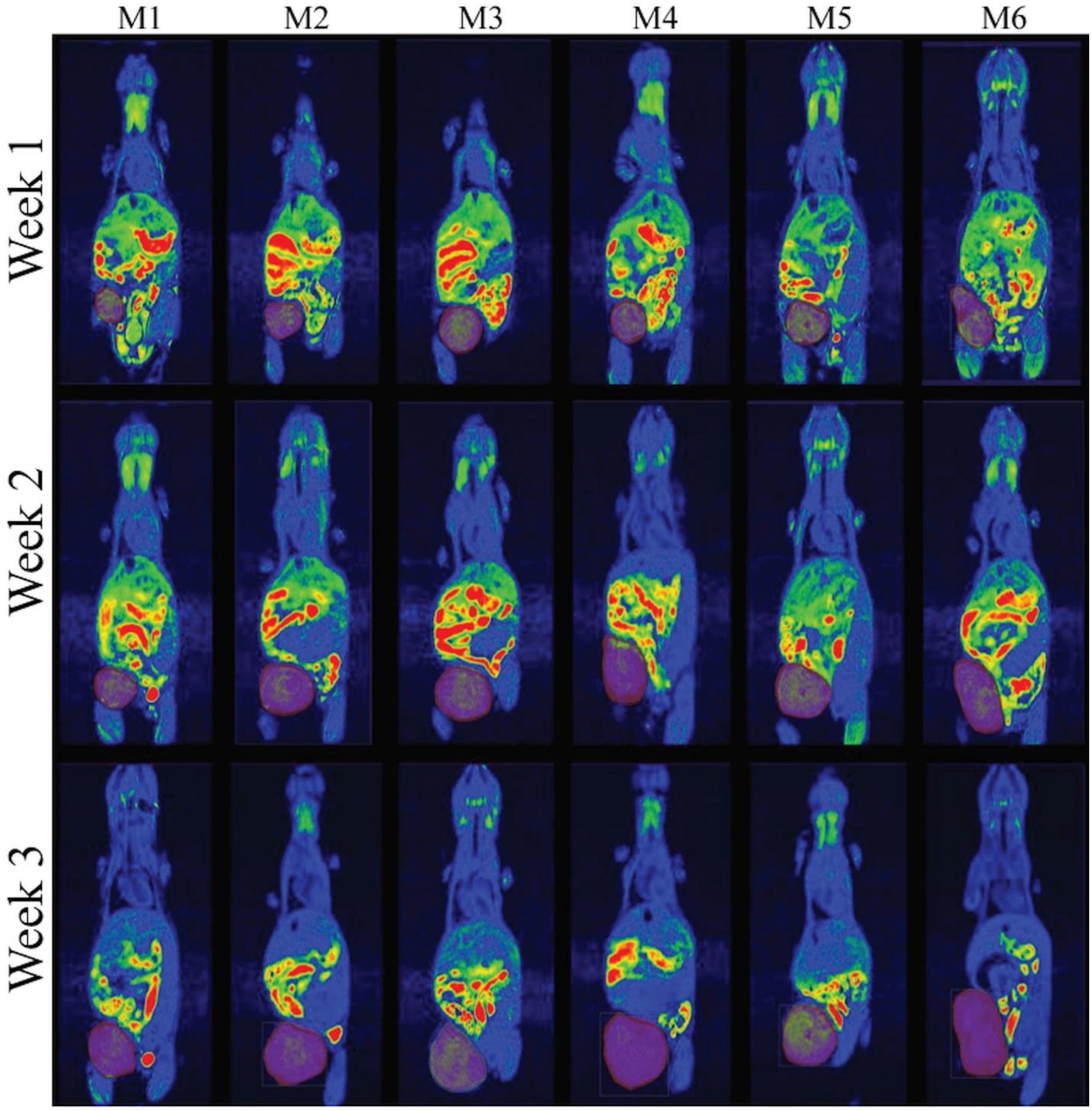

Figure S6. Weekly evaluations by MRI of each tumor bearing animals treated with Gadovist.

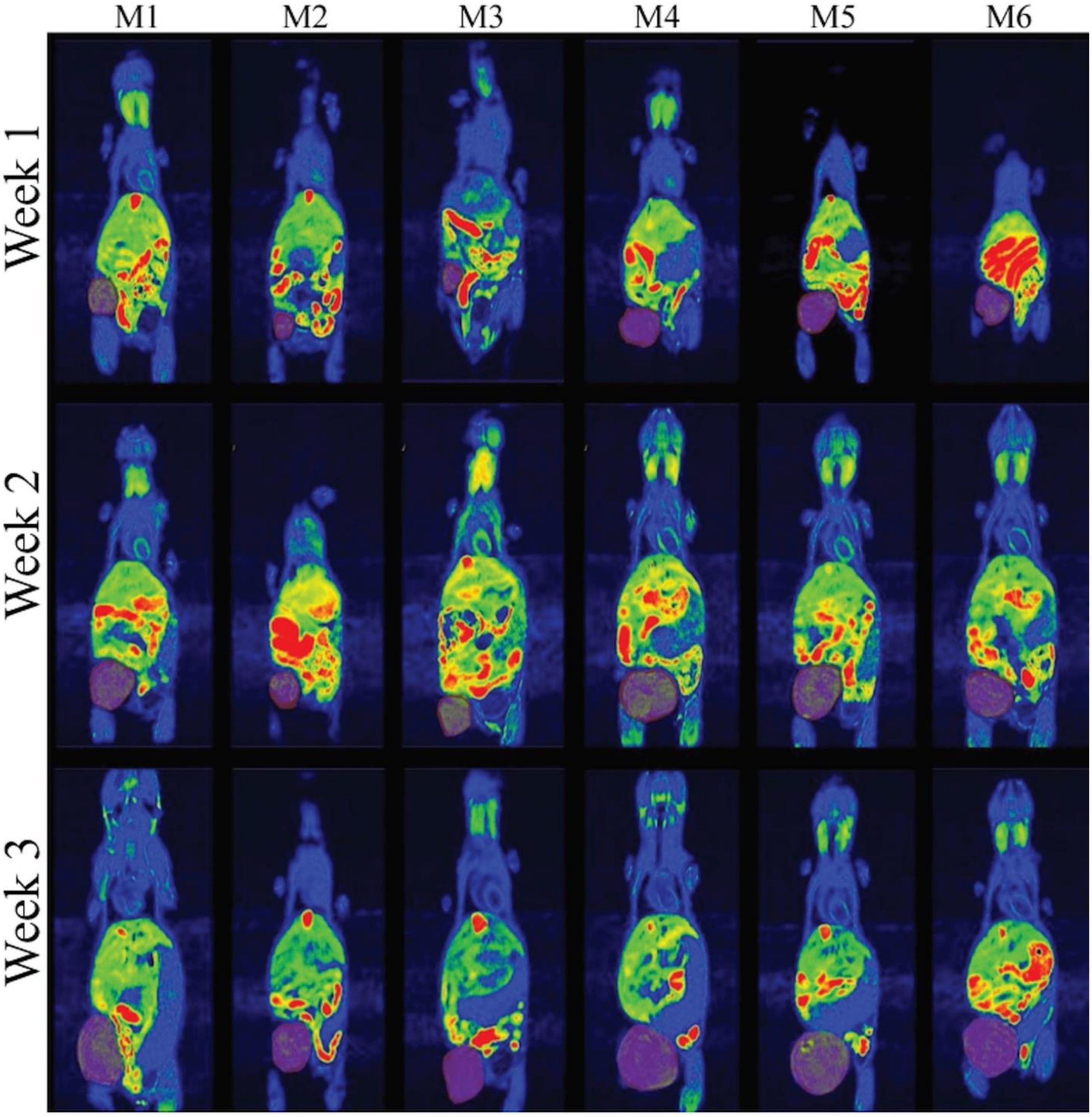

Figure S7. Weekly evaluations by MRI of each tumor bearing animals treated with Mn-CDs-NHF

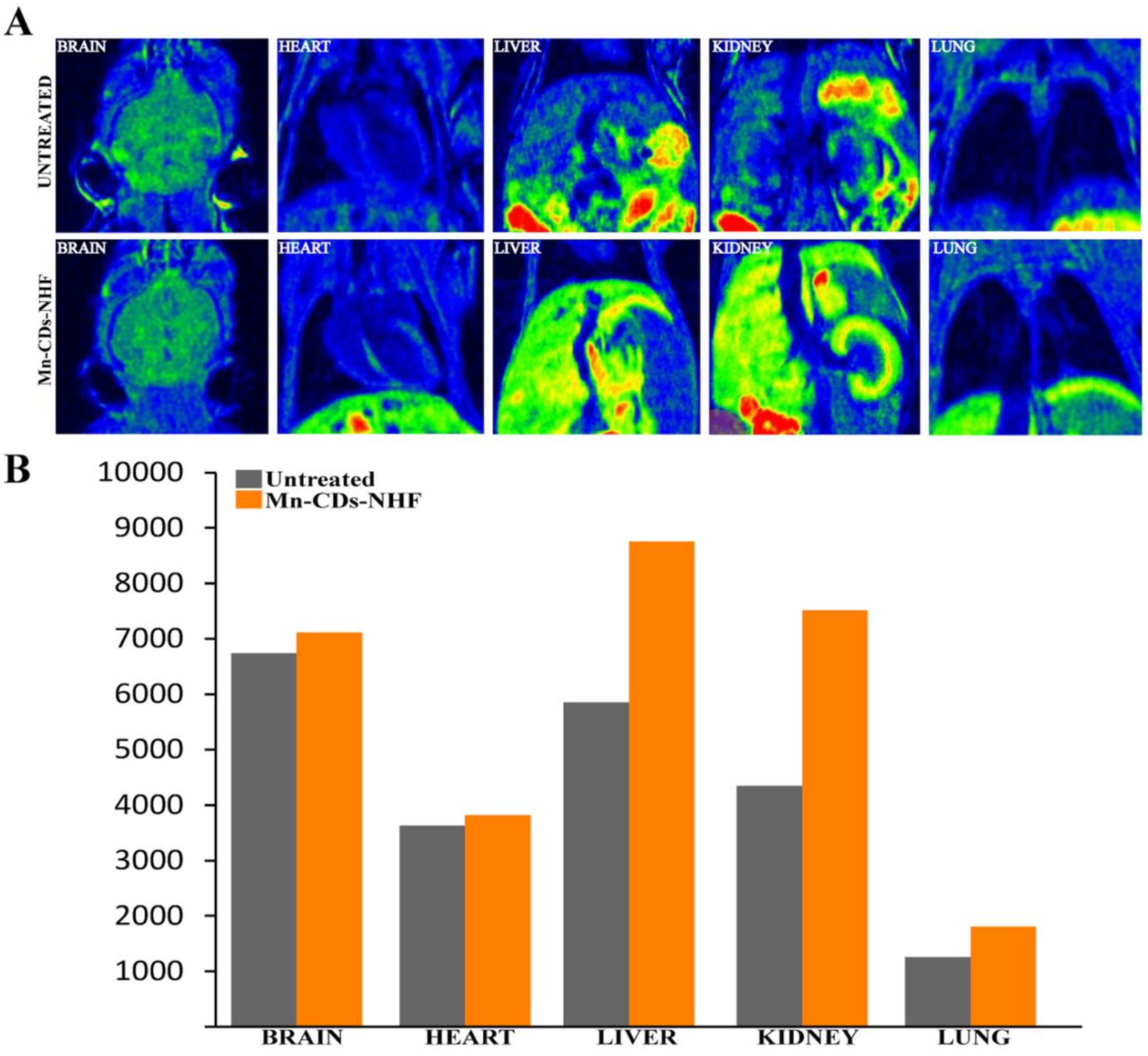

Figure S8. In vivo tissue distribution of Mn-CDs-NHF. A. Representative MRI images; B. MRI signal intensity.

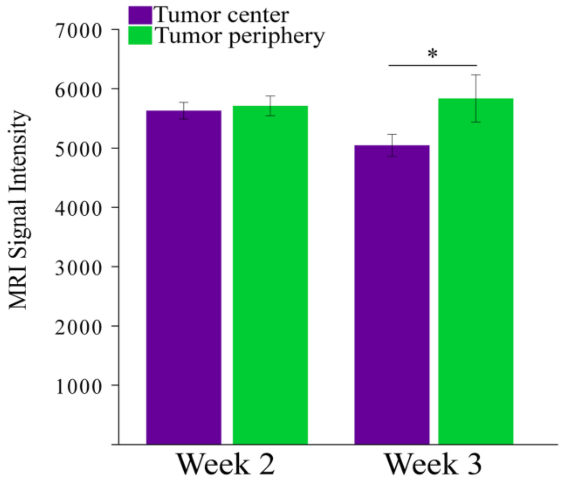

Figure S9. Quantification of MRI signal intensity in primary tumors. p=0.03 (paired t-test).
